# Ginsenoside Rb1 does not halt osteoporotic bone loss in ovariectomized rats

**DOI:** 10.1371/journal.pone.0202885

**Published:** 2018-09-13

**Authors:** JiaXin Bei, XinLe Zhang, JingKai Wu, ZhuoQing Hu, BiLian Xu, Sien Lin, Liao Cui, Tie Wu, LiYi Zou

**Affiliations:** 1 Department of Pharmacology, Guangdong Medical University, Zhanjiang, China; 2 Guangdong Key Laboratory for Research and Development of Natural Drugs, Guangdong Medical University, Zhanjiang, China; 3 Research Center of CoQ10, Guangdong Runhe Biochemical Technology Company, Dongguan, China; Mayo Clinic Minnesota, UNITED STATES

## Abstract

Osteoporosis (OP) is a systemic skeletal disorder, manifesting with a reduction in bone mass and deterioration of the microarchitecture. Mesenchymal stem cells (MSCs) have an innate ability to differentiate into several cell types, including osteoblasts (OB). Ginsenoside Rb1 (GRb1) is an ethanol extract from ginseng and contains a highly concentrated form of ginsenoside. GRb1 shows extensive beneficial health effects such as anti-oxidative and anti-inflammatory functions, modulating the immune system and inhibiting osteoclastogenesis. We hypothesized that GRb1 can promote MSC differentiation into OBs and inhibit bone loss. In the present study, we aimed to address two questions: (1) Will GRb1 have a positive effect on osteogenic differentiation of MSCs? and (2) Will GRb1 halt bone loss in ovariectomized (OVX) rats? We investigated the effects of GRb1 on viability and osteogenic differentiation of rat mesenchymal stem cells (rMSCs). Our results showed that GRb1 at concentrations of 10^−8^ M and 10^−6^ M can increase alkaline phosphatase activity, mineralization and the expression of osteogenic related proteins, such as osteopontin and osteoprotegerin, while incubating rMSCs with osteogenic induction medium and GRb1. Adding GRb1 into the medium can prevent rMSCs from Oxidative damage at the concentration of 25μM H_2_O_2_. Furthermore, 40 4-month-old rats were assigned to 5 groups(8 rats per group): the basal group, the sham group, the OVX group, the high dose of GRb1 group (6 mg/kg/day) and the low dose of GRb1 group (3 mg/kg/day). Rats recrived treatment 3days after surgery and last for 14 weeks. Examinations included serum analysis, mechanical testing, Masson-Goldner trichrome staining and bone histomorphometry analysis. The results showed that OVX can lead to dyslipidemia and excessive oxidative stress, whereas GRb1 cannot significantly halt dyslipidemia and excessive oxidative stress in OVX rats. In addition, the bone density of the lumbar vertebra and femur were decreased significantly in the OVX rats, and GRb1 could not inhibit bone loss. Bone histomorphometry analysis showed that the number and width of bone trabecula of the tibia were reduced in OVX rats, and GRb1 could not prevent their occurrence. A bone biomechanics assay showed that GRb1 cannot improve the ability of bone structure to resist fracture of the femur in OVX rats. The current study demonstrated that GRb1 has an obvious effect on osteogenic differentiation in rMSCs but no obvious effect on bone loss in OVX rats. These findings indicate GRb1 has a positive effect on rMSCs but does not have an effect on bone loss in OVX rats at the concentration we used.

## Introduction

Osteoporosis (OP) is a systemic skeletal disorder, manifesting as a reduction of bone mass and deterioration of microarchitecture, which may lead to osteoporotic fracture [1]. Bone mass is a dynamic equilibrium associated with bone formation mediated by osteoblasts and bone resorption mediated by osteoclasts. The balance is associated with many factors such as hormones, cytokines and mechanical stimulation [1–2]. Estrogen deficiency occurring after menopause activates both osteoclasts and osteoblasts, with activation of the former being dominant, resulting in a decrease in bone mass [3]. There is a subtle relationship between osteoporosis and lipid metabolism. Yamaguchi et al [4] investigated the association between blood lipid levels and osteoporosis in 214 postmenopausal women. The results suggest that blood lipid levels are closely related to bone mass and bone fragility. Osteogenesis in bone marrow is related to adipogenesis. Bone marrow stromal cells (MSCs) prefer to differentiate into adipocytes under peroxisome proliferators-activated receptors (PPARγ)-mediated while osteoblast differentiation is inhibited. PPARγ mainly exists in adipose tissue. Immunohistochemical experiments also detected the presence of PPARγ protein in fat-rich bone marrow[5]. Disorders of fat metabolism accelerate OP. Currently, the most prescribed drugs against OP, such as calcium, vitamin D, bisphosphonates, raloxifene, PTH, hormone replacement and calcitonin, are effective in preventing bone resorption or promoting bone formation. However, these drugs do not address the other pathological changes that accompany OP. In particular, they neglect its detrimental effects on bone marrow lipid metabolism and the micro-circulation system [6–7]. Meanwhile, it has been reported that many Chinese herbal medicines have an active effect on OP [8–12].

Mesenchymal stem cells (MSCs) reside in mesenchymal tissues, i.e., bone marrow, adipose, umbilical cord and the peripheral blood [13–15]. MSCs can differentiate into several cell types, such as bone cells (osteoblasts), cartilage cells (chondrocytes), muscle cells (myocytes), fat cells (adipocytes), myoblasts and neurons [16–17]. The process of MSCs differentiating into mature osteoblasts is very complex and regulated by many transcriptional factors, signaling pathways and micro RNAs[18–21]^.^

Ginseng, the root of Panax ginseng C.A. Meyer, has been used as a tonic in clinical applications in China for more than 2000 years. It has been found that ginsenosides (GSS) contain more than 30 different compounds that have been identified as active components [22]. Ginsenoside Rb1 (GRb1) is an ethanol extract from ginseng and contains a concentrated solution of many different ginsenosides (GSS). According to previous studies, GRb1 shows wide beneficial health effects in animals, including anti-cancer[23], anti-oxidative stress[24], and anti-inflammatory[25] effects; modulation of the immunological system[26]; increased basal glucose uptake[27]; neuroprotection; nephroprotection[28], and inhibition of osteoclastogenesis[29]. It has been reported that GRb1 may ease osteoporosis by regulating the expression of osteoprotegerin (OPG), a receptor activator of NF-κB ligand (RANKLe) in vitro experiment[30]. In addition, GRb1 promotes MSC differentiation towards mature functional osteoblasts by enhancing Runt-related transcription factor 2 (Runx2) expression [29].

However, few studies have investigated the changes in bone metabolism resulting from the effect of GRb1 on osteogenic differentiation. In particular, for estrogen deficiency-induced osteoporosis, does GRb1 inhibit bone loss in ovariectomized rats or affect bone metabolism in ovariectomized rats? We have investigated the effect of GRb1 on osteogenic differentiation of rat MSCs (rMSCs). Meanwhile, we explored the actions of GRb1 on bone mass and density in ovariectomized rats. Our results show that GRb1 promotes osteogenesis of MSCs but it does not have any effect on bone loss in ovariectomized rats.

## 2 Materials and methods

### 2.1 Cell culture

All of the cells were isolated from a 4-week-old male Sprague-Dawley (SD) rat. The details of rBMSC isolation and culture have been described previously. Briefly, the cells were cultured in alpha modified Eagle’s medium (a-MEM, Gibco) supplemented with 10% fetal bovine serum (FBS, Gibco), 100 U/ml penicillin and 100 mg/ml streptomycin (Gibco) at 37°C in a 95% humidified atmosphere of 5% CO_2_. Surface markers including CD34, CD44, CD45, and CD90 (BD Biosciences) were used to determine the purity of MSCs. We used rBMSCs of passages 3–6 in the experiments.

### 2.2. Cell viability assay

Cells (2 × 10^3^ per well) were subcultured in a 96-well plate. After 24 h of incubation, the medium was changed to media containing GRb1 (PureChem-Standard, Chengdu, China) at different concentrations (10^−4^ to 10^−10^ mol/L). The cells were incubated at 37°C for 24 to 72 h.

The cellular proliferation was determined using a methyl thiazolyl tetrazolium (MTT) assay. Briefly, cells were treated with the MTT solution (final concentration, 0.5 mg/ml) for 4 h at 37°C. The dark-blue formazan crystals formed in intact cells were solubilized with 150 μL of DMSO, and the plate was shaken for 10 min. The absorbance at 570 nm was measured with a microplate reader.

### 2.3. Anti-oxidative effect assay

rMSCs were incubated with GRb1 at a concentration of 10^−6^ mol/L for 24 h and then further incubated with H_2_O_2_ at different concentrations (25, 50, 100, 150, 200, 250, 300, 350, or 400 μM) for another 24 h. Then an MTT assay was performed to measure whether GRb1 has an anti-oxidative effect.

### 2.4. Alkaline phosphate activity assay

After the MSCs were incubated with or without osteogenic induction medium (OIM, 1 nM dexamethasone, 50 mM L-ascorbic acid-2-phosphate, and 20 mM b-glycerophosphate with complete medium) and GRb1 (10^−6^ or 10^−8^ mol/L) for 7 days, they were subjected to an alkaline phosphate (ALP) assay. Briefly, the cells were washed with PBS and then fixed with 70% ethanol. Then, ALP substrate solution (BCIP-NBT) was added to the cells and incubated in the dark. Finally, DMSO was added to the ALP-stained plate, and the absorbance at 540 nm was measured to estimate ALP activity.

### 2.5. Mineralization assay

After 7 to 14 days of osteogenic induction, the cells were fixed with 70% ethanol for 10 min. Then, the fixed cells were stained with 0.5% Alizarin red S (pH 4.1) for 10 min at room temperature and washed three times with deionized water. Orange red staining indicated the position and intensity of calcium deposits. The calcium deposition was extracted with 10% cetylpyridinium chloride (CPC, Sigma) and quantified by measuring the absorbance of the extract at 550 nm.

### 2.6. RNA extraction and real-time PCR

Total RNA was extracted from cultured cells with an RNeasy Mini Kit (Qiagen, USA), and first-strand cDNA was synthesized using M-MLV reverse transcriptase (Promega, USA) according to the manufacturer’s instructions. Real-time PCR was performed using the Step One Plus Real-Time PCR System (Applied Biosystems, USA). The reaction conditions consisted of 15-μl reaction volumes with 3 μL of diluted cDNA template, 7.5 μl of SYBR-Green Master Mix (2×), 3.9 μL of PCR-Grade water and 0.3 μL of each primer (10 μM). Amplification conditions were as follows: first, 95°C for 5 min and then 40 cycles at 95°C for 15 s and 60°C for 60 s. Primer sequences were as follows: osteopontin (OPN) forward: 5’-GTACCCTGATGCTACAGACG-3’, reverse: 5’-TTCATAACTGTCCTTCCCAC-3’; Runt-related transcription factor (Runx2) forward: 5’-ACTTCCTGTGCTCGGTGCT-3’, reverse: 5’-GACGGTTATGGTCAAGGTGAA-3’; osteoprotegerin (OCN) forward, 5'-GCAGCTTGGTGCACACCTAG-3' and reverse, 5'-GGAGCTGCTGTGACATCCAT-3'; ALP forward, 5'-GACTGGTACTCGGATAACGAGA-3' and reverse, 5'-CTCATGATGTCCGTGGTCAATC-3'.

### 2.7 Animals

Forty 4-month-old specific pathogen free (SPF) grade Sprague-Dawley rats were raised in an SPF criterion animal feeding room. Twenty-four rats were subjected to bilateral ovariectomy surgery (OVX) under anesthesia. The OVX rats were intraperitoneally injected once a day, with 9% sodium chloride solution as the vehicle (n = 8) or with GRb1 (6 mg/kg/day, once a day, n = 8 or 3 mg/kg/day, once a day, n = 8) dissolved in the vehicle. Rats started to receive the treatment 3 days after the ovariectomy surgery. The animal drug concentration was calculated according to the cell concentration. Plasma concentrations of drugs in rats are approximately the same as drug concentrations in culture medium.

Eight rats were sham operated under anesthesia and intraperitoneally injected with vehicle once a day. The remaining 8 rats were euthanized on the day of surgery as basal controls. The rats were treated for 12 weeks until euthanasia. On the 13^th^, 14^th^, and 3^rd^ and 4^th^ day before euthanasia, all of the rats were subcutaneously injected with 0.7% calcein. The tibia, femur, lumbar vertebrae and blood were harvested. The left tibia was cut into distal and proximal sections then fixed in 10% neutral formalin and embedded in 70% ethanol for bone histomorphometry measurement. After wrapping the right femur with gauze soaked in saline, wrap it in foil and put it in a sealed bag, finally stored them at -80°C.

Before the mechanical test for bone biomechanics measurement, the femurs were thawed at room temperature. The femurs were stored in 0.9% saline solutions while undergoing the bone biomechanics and bone density tests.

Animal experiments were carried out in strict accordance with the recommendations in the Guide for the Care and Use of Laboratory Animals of Guangdong Laboratory Animal Monitoring Institute under the National Laboratory Animal Monitoring Institute of China. Animal experiments were approved by Guangdong Medical University Experimental Animal Ethics Committee (Approval number:GDY1602009). 0.13ml/100g of 3% pentobarbital injection were intraperitoneal injected for ovarian surgery and euthanasia. And all efforts were made to minimize suffering.

### 2.8 Body weight, serum markers assay and observations of pathological section of the uterus

The rats were weighed every week. At the end of the experiments, the rats were sacrificed by cardiac puncture under anesthesia. Their uteruses were removed and weighed. The uteruses were fixed in 10% neutral formalin then embedded in paraffin. Sections of 5 μm were cut by a slicing machine for HE staining. The blood was centrifuged at 3000 rpm at 4°C for 10 mins, and the supernatant was carefully transferred to another tube and stored at −80°C until analysis. The level of triglycerides (TG), total cholesterol (TC), high density lipoprotein (HDL), low density lipoprotein (LDL), glutathione (GSH) and superoxide dismutase (SOD) in the serum of the rats was measured by using a Triglycerides Assay Kit, Total Cholesterol Assay Kit, High-Density Lipoprotein Cholesterol Assay Kit, Low-Density Lipoprotein Cholesterol Assay Kit, Reduced Glutathione Assay Kit, and Superoxide Dismutase Assay Kit (WST-1 method) (Nanjing Jiancheng Bioengineering Institute, Nanjing, China). Absorbance (the reading at 450 nm or 560 nm) of each biochemical marker was read and compared across the groups.

### 2.9 Bone histomorphometry, bone biomechanics and bone mass density

The left tibia was cut into distal and proximal sections then fixed in 10% neutral formalin and put into 70% ethanol for 24h, then put them into 80% ethanol for 12h, then 85% ethanol for 6h, then 90%,95%,100% ethanol till samples were desiccated. Methyl phenylacetate was used to embed the upper and middle segments of the humerus. And the proximal segment of the bone-embedded block was cut to a thickness of 5 μm, and the distal segment of the bone-embedded block was cut to a thickness of 8 μm by slicer machine. Bone histomorphometry is a type of measurement used for evaluating bone architecture and bone mass by testing static parameters via a digitized image analysis system (Osteometrics, Inc. Decatur, GA, USA), such as percent trabecular area (%Tb.Ar), trabecular width (Tb.Wi), trabecular number (Tb.N), trabecular separation (Tb.Sp), cortical area (Ct.Ar), percent cortical area (%Ct.Ar), percent marrow area (%Ma.Ar), labeled perimeter (%L.Pm) and so on. A digitizing analysis image was used on a 5-μm section of a longitudinal section of the proximal tibia and an 8-μm section of a transverse section of the distal tibia for histomorphometric measurements. For the proximal tibia, the region of interest was located between 1 and 3 mm distal to the growth plate–epiphyseal junction.

The right femurs were subjected to a dual energy X ray test to measure the BMD of the whole femur, distal femur and proximal femur. The same X-ray test was performed on the fourth lumbar vertebrae (The DXA energy setting: point resolution 0.0622cm, 140/100kVp, 2.5 mA avg, 179 seconds, 50Hz). And then the right femurs were used to determine bone mechanical properties through a three-point bending test using a Bose Electro Force Testing System (ELF3510; Bose Corp., Eden Prairie, MN, USA). Bone samples were tested with a 1-mm indenter at a speed of 0.01 mm/s with a 15-mm span (L). Force (F) and deflection (D) were automatically recorded. The output parameters include yield load (N), ultimate load (N), and stiffness (N/mm). Finally got Maximum load, Break load, Elastic load and Siffness. The maximum load means the maximum load before fracture. The break load means the load when fracture happened. The Elastic load means the maximum load that the bone can withstand in the elastic range, representing the toughness of the bone. Stiffness indicates the structural strength of bone tissue.

### 2.10 Statistical analysis

All data are presented as the mean ± SD, and statistical analysis was performed using one-way analysis of variance (one-way ANOVA). The parametric assumptions of the one-way ANOVA were checked for validity and passed. A value of P < 0.05 was considered statistically significant.

## 3. Results

### 3.1 Effect of GRb1 on rMSCs viability and anti-oxidation

**Fig 1:**
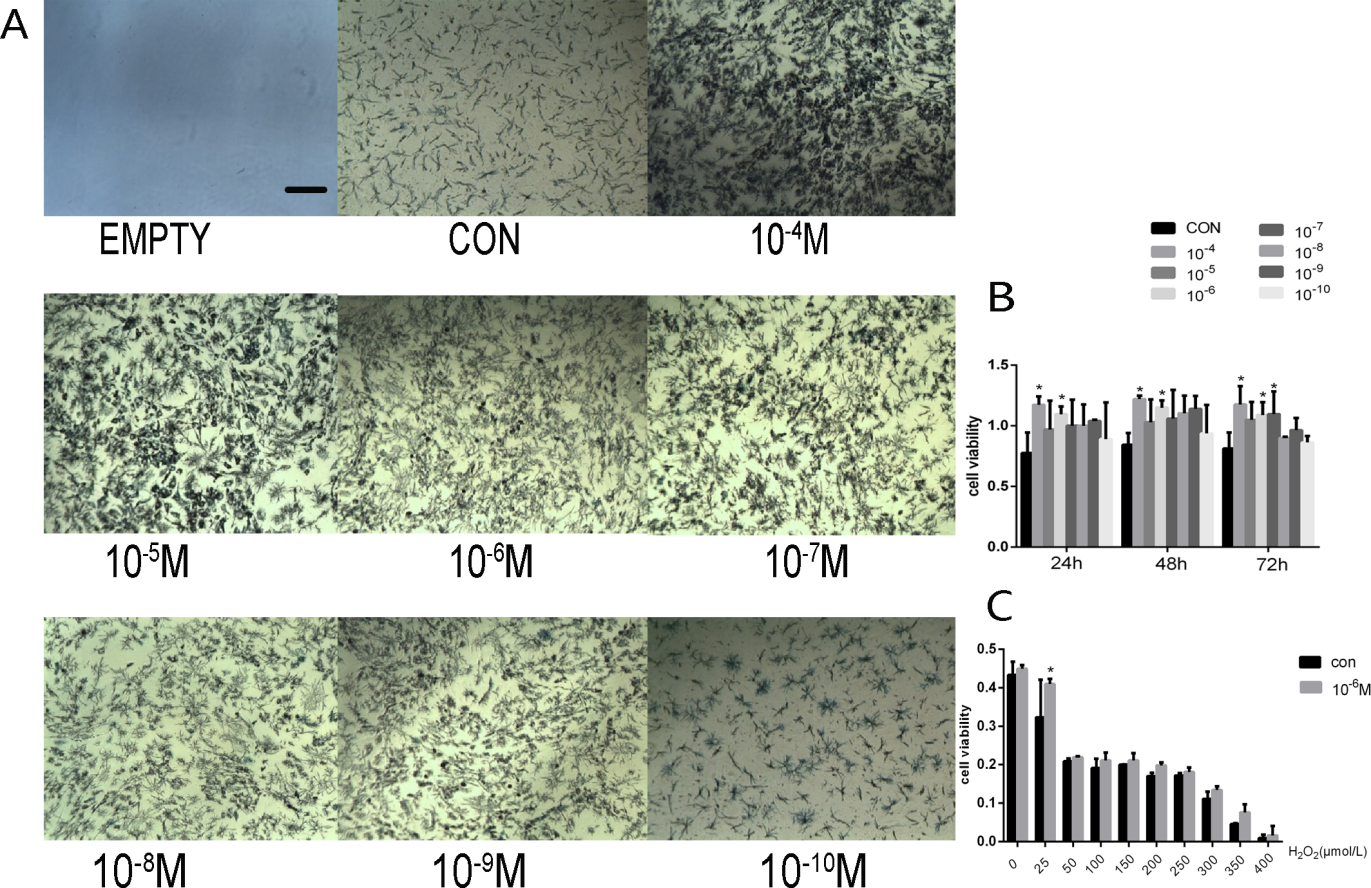
Effects of GRb1 on the viability of rMSCs. (A) rMSCs were incubated with GRb1 for 72 hours and the proliferation of rMSCs were captured under a microscope. (B) The viability of rMSCs treated with or without GRb1. (C) The effect of GRb1 on rMSCs treated with H2O2. *P < 0.05 compared with CON. Scale bar = 200 μm.

A MTT assay was performed to investigate the effect of GRb1 on rMSCs viability. The results showed GRb1 had a significant and positive effect on rMSCs at a dosage of 10^−4^–10^−7^ M (Fig 1A and 1B). The MTT assay was performed to investigate the anti-oxidative effect of GRb1 on rMSCs. GRb1 showed a good anti-oxidative effect on H_2_O_2_-induced rMSCs at a concentration of 25 μmol/l. As the concentration of H_2_O_2_ increased, the effect of GRb1 became non-significant (Fig 1C).

### 3.2 GRb1 promotes osteogenesis of rMSCs

**Fig 2:**
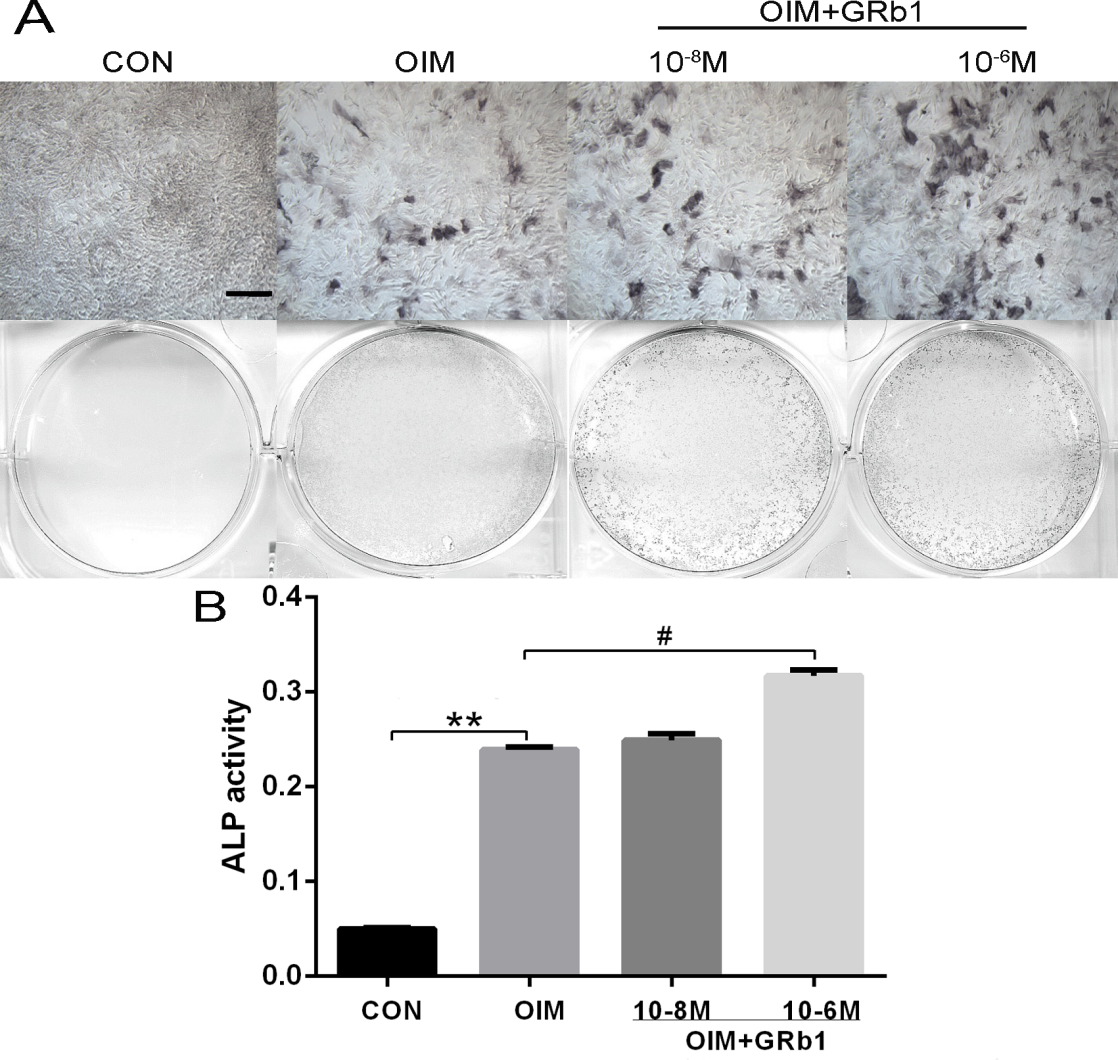
GRb1 increased ALP activity of rMSCs. (A) rMSCs were incubated with or without osteogenic medium and GRb1 for 7 days. (B) The ALP activity of rMSCs; *P < 0.05 compared with CON. #P < 0.05 compared with OIM. Scale bar = 200 μm.

Because ALP formation indicates the differentiation process of osteoblasts, ALP activity of rMSCs was measured after incubation with an increasing dose of GRb1 (10^−8^ M and 10^−6^ M) for 7 days. The data showed that ALP formation increased as the dose of GRb1 increased in a dose-dependent manner. GRb1 can significantly promote ALP activity at a concentration of 10^−6^ M (Fig 2).

**Fig 3:**
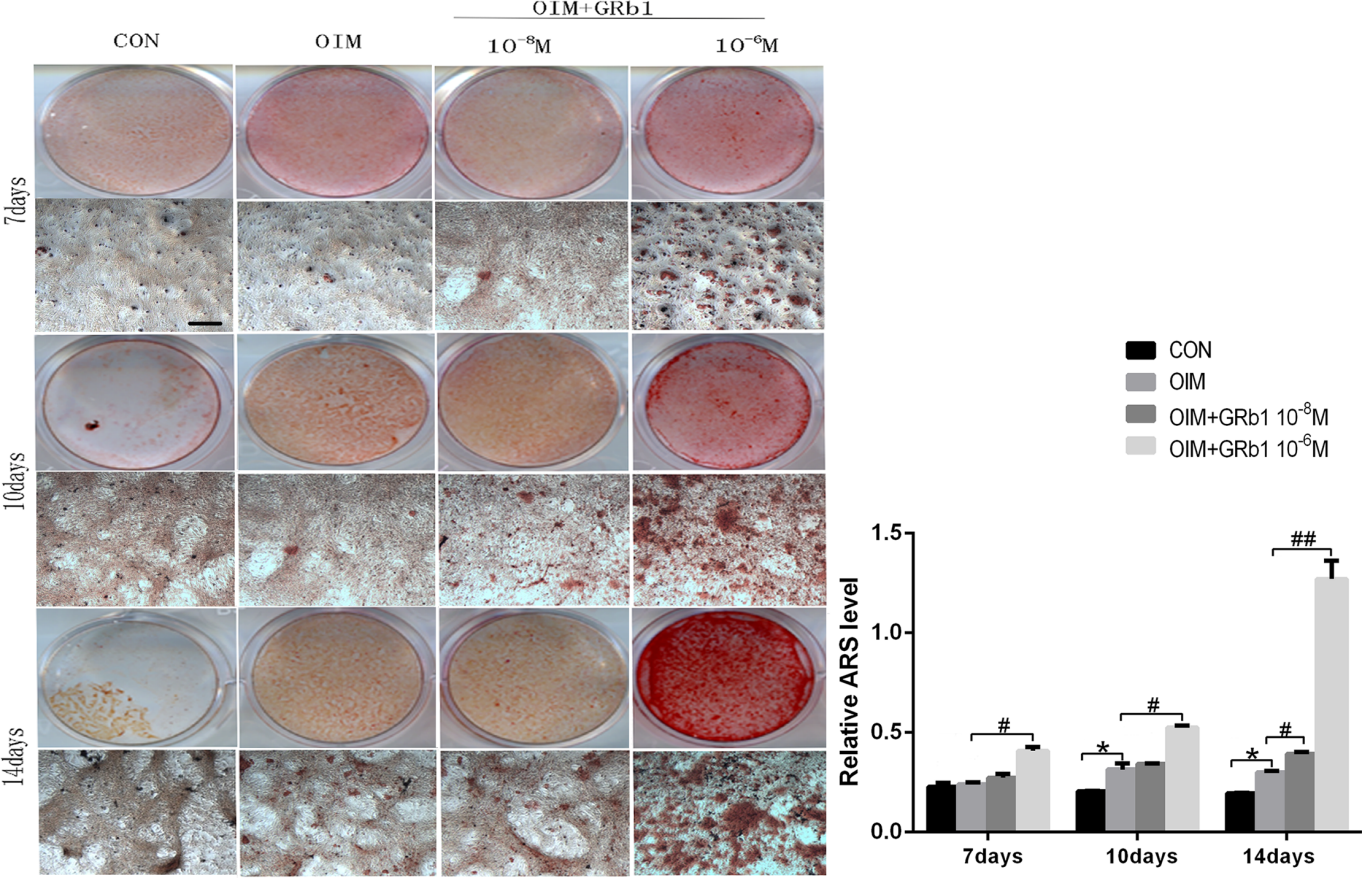
GRb1 promotes mineralization of rMSCs. (A) rMSCs were treated with or without osteogenic medium and GRb1 for 7, 10, or 14 days. (B) The calcium deposition was evaluated by extraction of Alizarin red S dye with 10% cetylpyridinium chloride. *P < 0.05 compared with CON. #P < 0.05,# #P < 0.01 compared with OIM Scale bar = 200 μm.

Furthermore, we examined the effect of GRb1 on calcium mineralization of rMSCs. Alizarin red S staining showed that there was no calcium nodule formation in the absence of OIM at day 7, while in the presence of OIM, 10^-6^M GRb1 can sharply increase calcium nodule formation, compared with OIM(Fig 3). On the 10^th^ day, rMSCs of OIM showed calcium nodule, while 10^-6^M GRb1 can sharply increase calcium nodule formation, compared with OIM(Fig 3). On the 14^th^ day, Both 10^-8^M GRb1 and 10^-6^M GRb1 can increase calcium nodule formation markerly(Fig 3). Quantification analysis of Alizarin red S staining analysis showed that GRb1 markedly increased calcium deposition in a concentration-dependent manner compared with the control, while the changes were most obvious at a concentration of 10^−6^ M (Fig 3).

**Fig 4:**
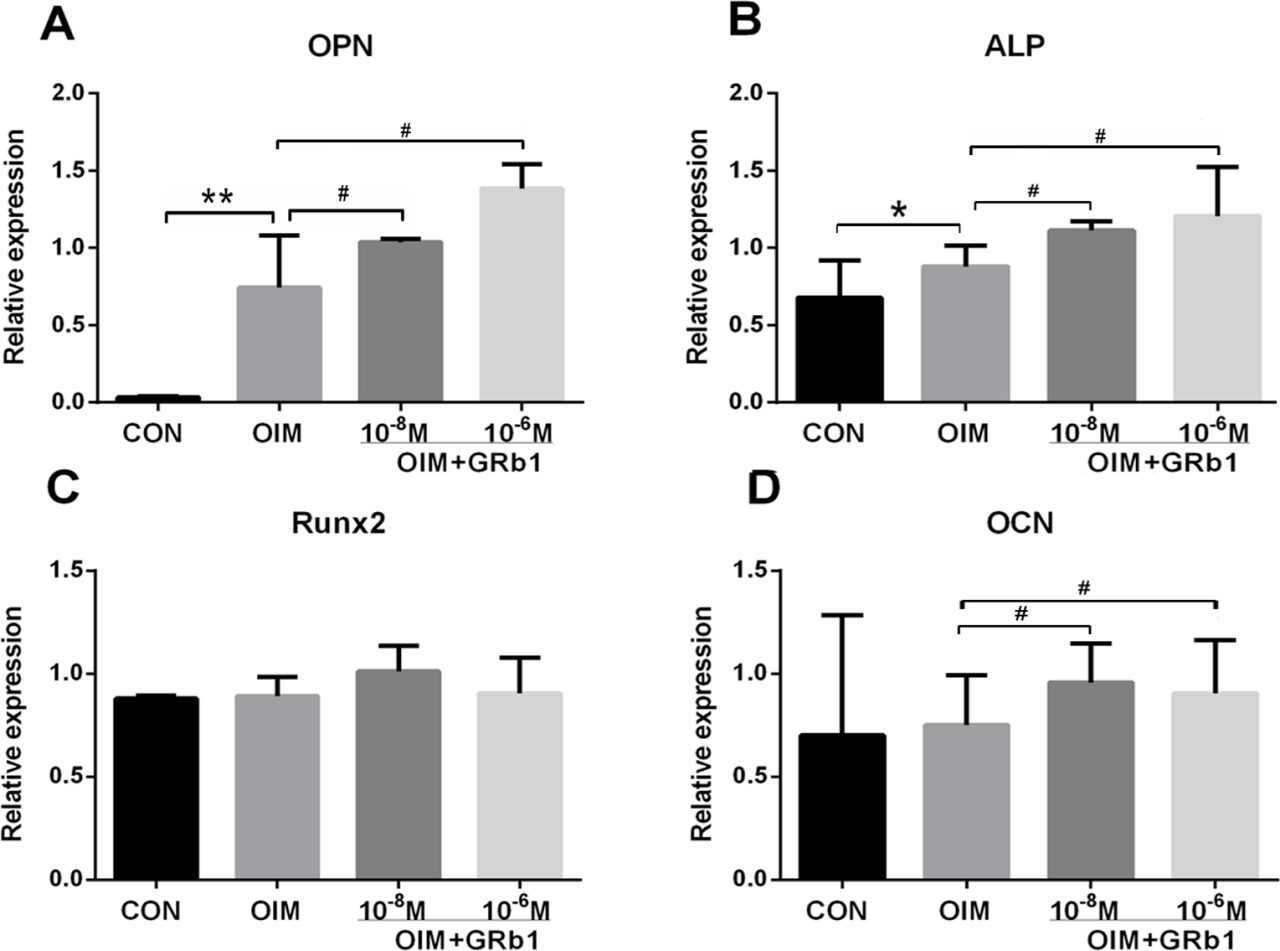
Effects of GRb1 on the expression of osteogenic-related genes of rMSCs. (A) The expression of OPN (B)The expression of ALP (C) The expression of Runx2 (D) The expression of OCN. *P < 0.05 compared with CON. # P < 0.05 compared with OIM.

Bone maturation is largely regulated by a series of bone matrix proteins in osteoblasts, such as oesteopontin (OPN), osteoprotegerin (OCN), ALP, and Runt-related transcription factor 2 (Runx2). OPN plays an important role in the process of attachment and mineralization of osteoblasts. We measured the mRNA expression of the bone matrix proteins mentioned above via real-time PCR. The results demonstrated that OPN and ALP were both significantly up-regulated (P < 0.05) in a dose-dependent manner compared with the control and with OIM (P < 0.05). However, the expression levels of Runx2 and OCN were not markedly changed. Therefore, we concluded that GRb1 could promote the expression of OPN and ALP to encourage osteoblastic differentiation (Fig 4).

### 3.3 The effect of GRb1 on weight, the uterus and blood

**Fig 5:**
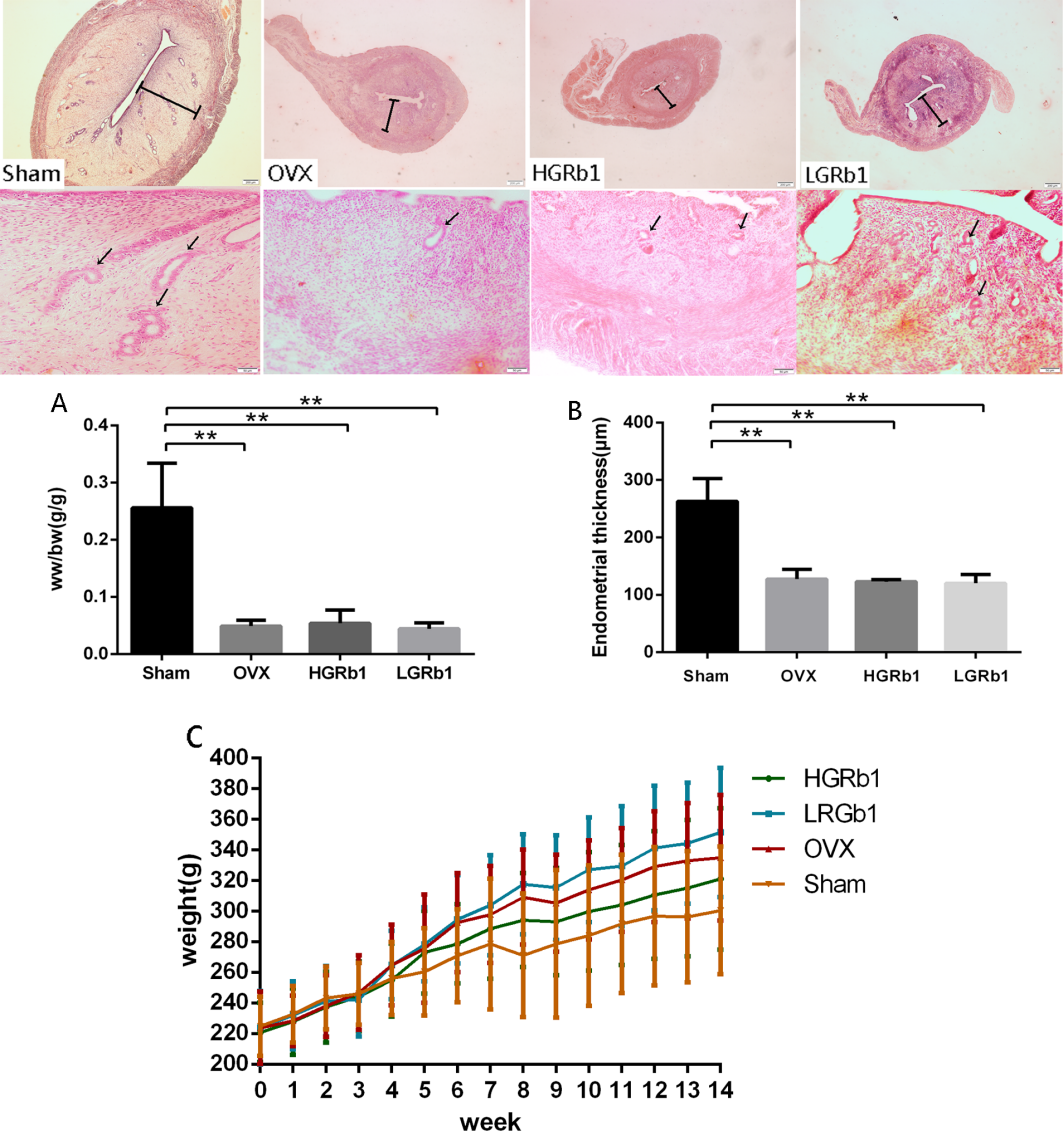
The effect of GRb1 on weight and uterus related parameters. (A) The proportion of weight of the uterus (B) The thickness of the endometrium (C) The trend chart of the average weight of each group. *P < 0.05 **P < 0.01 compared with Sham.

Increasing weight is a typical characteristic of OVX rats because of an accumulation of fat. To determine whether GRb1 could reduce the weight of OVX rats, we weighed each rat weekly and compared the data among groups. The results showed that the rats’ weights increased significantly (P < 0.05) as time passed, but differences in weights across each group did not obviously change every week. However, the tendency in the high-dose (6 mg/kg) GRb1 group was a gradual slowing of weight gain (Fig 5C). GRb1 can prevent OVX rats from rapid gaining weight. The thickness of the endometrium was obviously decreased (P < 0.05) (Fig 5B) compared with the sham, and the GRb1-treated rats’endometrium were not different from the OVX ones. Additionally, the proportion of the weight of the uterus and body weight did not change in the GRb1-treated and OVX groups, while the OVX decreased significantly (P < 0.05) compared with the sham (Fig 5A). From the stained images, the endometrial glands of the OVX rats were reduced compared to the sham group, and the endometrial glands were increased after the GRb1 administration. Also, we can see the lipid metabolism in tibia were disturbed after ovariectomy, and GRb1 cannot halt it (S1 Fig).

Triglyceride (TG), total cholesterol (TC), high density lipoprotein (HDL) and low density lipoprotein (LDL) were used to evaluate the levels of fat in the blood. After ovariectomy, the four indices of blood fat in the rats increased significantly, but rats treated with GRb1 did not present a decreasing trend, except for HDL, which hinted the estrogen-deficiency model was successful but that GRb1 cannot block the deteriorating index of blood fat at the concentration we used.

Estrogen deficiency induced bone loss and presented with an accumulation of oxidative stress. Glutathione (GSH) and superoxide dismutase (SOD) can remove oxygen free radicals and protect against oxidative stress[29]. GSH and SOD levels in the serum were measured to investigate the effect of GRb1 on OVX rats. GRB1 had little effect on blood fat and oxidative stress in OVX rats at concentrations of 3 mg/kg and 6 mg/kg. SOD and GSH in OVX rats decreased significantly (P < 0.05). GRb1 cannot alter the change of SOD and GSH in OVX rats, but the results of HGRb1 (high dose of GRb1 group) and LGRB1 (low dose of GRb1 group) presented an increasing trend in SOD (S2 Fig).

### 3. 4 The effect of GRb1 on bone loss in OVX rats

**Fig 6:**
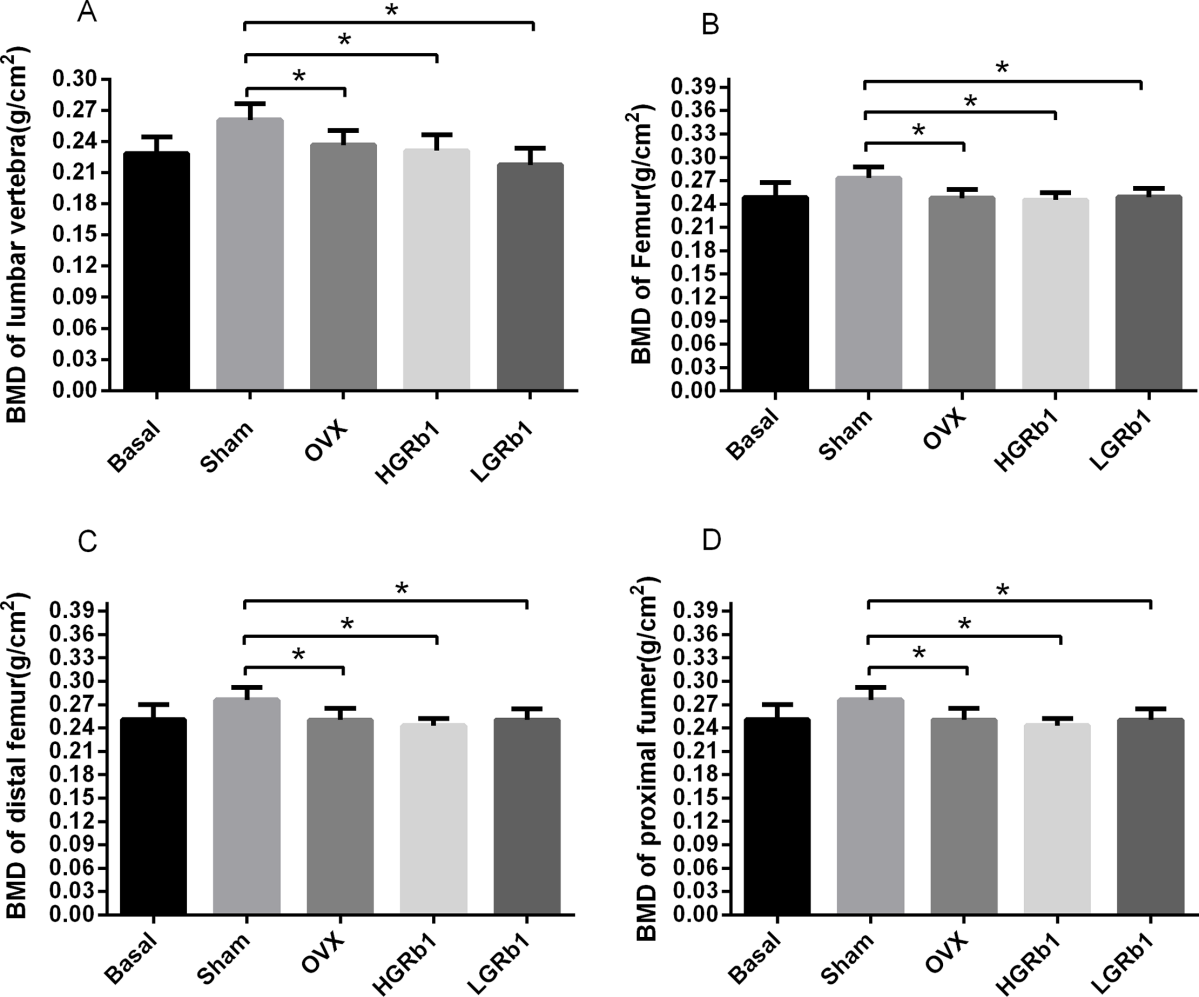
BMD tests were performed on lumbar vertebrae (LV) and femur. (A) BMD of LV (B) BMDs of the femur (C) BMD of the distal femur (D) BMD of the proximal femur. *P < 0.05, **P < 0.01 compared with Sham.

The abundant loss of bone mineral density is one of the diagnostic criteria for osteoporosis. Rats treated with ovariectomy present with serious bone loss, including decreases in the amount and mineral density of bone. The results of BMD tests showed that, compared with the sham group, the BMD of lumbar vertebrae and femurs in OVX rats were remarkably reduced (P < 0.05) (Fig 6). It is noteworthy that the BMD of the proximal femur and the distal femur decreased significantly in OVX group, when compared with the sham group (P < 0.01). After treatment with GRb1 for 12 weeks, there were no changes. GRb1 had no effect on BMD at a concentration of 3 or 6 mg/kg.

Maximum load, break load, elastic load and stiffness were obtained via a bone biomechanics test, which aimed to evaluate intensity and the weight-bearing function of the femur, separately representing the load at break, the maximum strength that can be sustained, the external force needed for bone deformation and the ability of bone structures to resist fracture. Maximum load, break load and stiffness were significantly decreased (P < 0.05) compared with the sham group, while no significant difference was found for the elastic load. In addition, the results of the GRb1 groups were not improved (S3 Fig).

**Fig 7:**
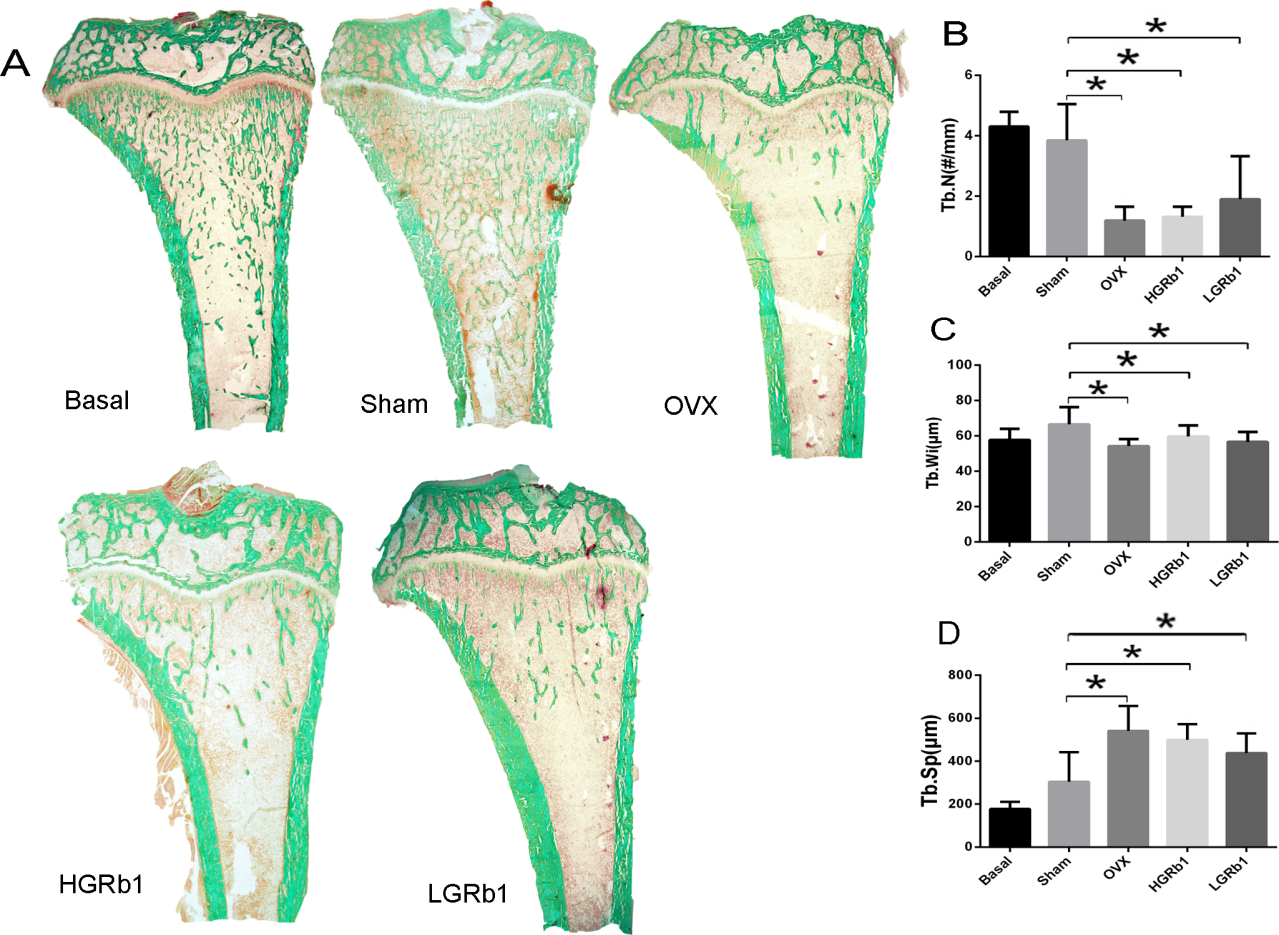
Representative proximal tibia fluorescence micrographs and histomorphometry analysis. (A) The 5 μm longitudinal section of the proximal tibia with Masson-Goldner Trichrome staining. (B) Tb.N (C) Tb.Wi (D) Tb.Sp. *P < 0.05 compared with Sham.

We also measured the dynamic parameters, such as percent labeled perimeter (%L.Pm), mineral apposition rate (MAR), and bone formation rate (BFR) based on fluorescence labeling and tissue staining to observe the changes in the characteristics of the bone, including the bone mass and the quality of the bone. The number of tibia(Tb.N)and the width of tibia(Tb.Wi) of OVX rats were much lower (P < 0.05) (Fig 7B and 7C) than those of the sham group, while Tb.Sp (Fig 7D) was remarkably increased (P < 0.05). There was a significant difference in bone mass and bone width between the OVX and sham groups. After treatment with GRb1, Tb.N, Tb.Wi and Tb.Sp were not improved.

Based on these results, we know that OVX can damage the morphological structure of trabecular bone. Unfortunately, GRb1 cannot improve the situation at doses of 6 and 3 mg/kg. Furthermore, %Tb.Ar is an objective standard used to evaluate bone mass. OVX decreased %Tb.Ar sharply (P < 0.05) compared with the sham group (S4 Fig). However, bone formation can be stimulated under estrogen deficiency, because the data show that MAR and %L.Pm, mirroring the dynamic state of bone growth, were enhanced significantly (P < 0.01, P < 0.05) (S4 Fig). In addition, there were significant elevations (P < 0.05) (S4 Fig) in bone formation rate (BFR/BV and BFR/TV) (S4 Fig) but significant reductions (P < 0.05) (S4 Fig) in BFR/BS because estrogen deficiency-induced bone loss is much more serious than stimulated bone growth. Unfortunately, our results show that GRb1 did not inhibit bone loss from the proximal tibia in OVX rats, regardless of the morphological structure of trabecular bone or the dynamic state of bone growth.

**Fig 8:**
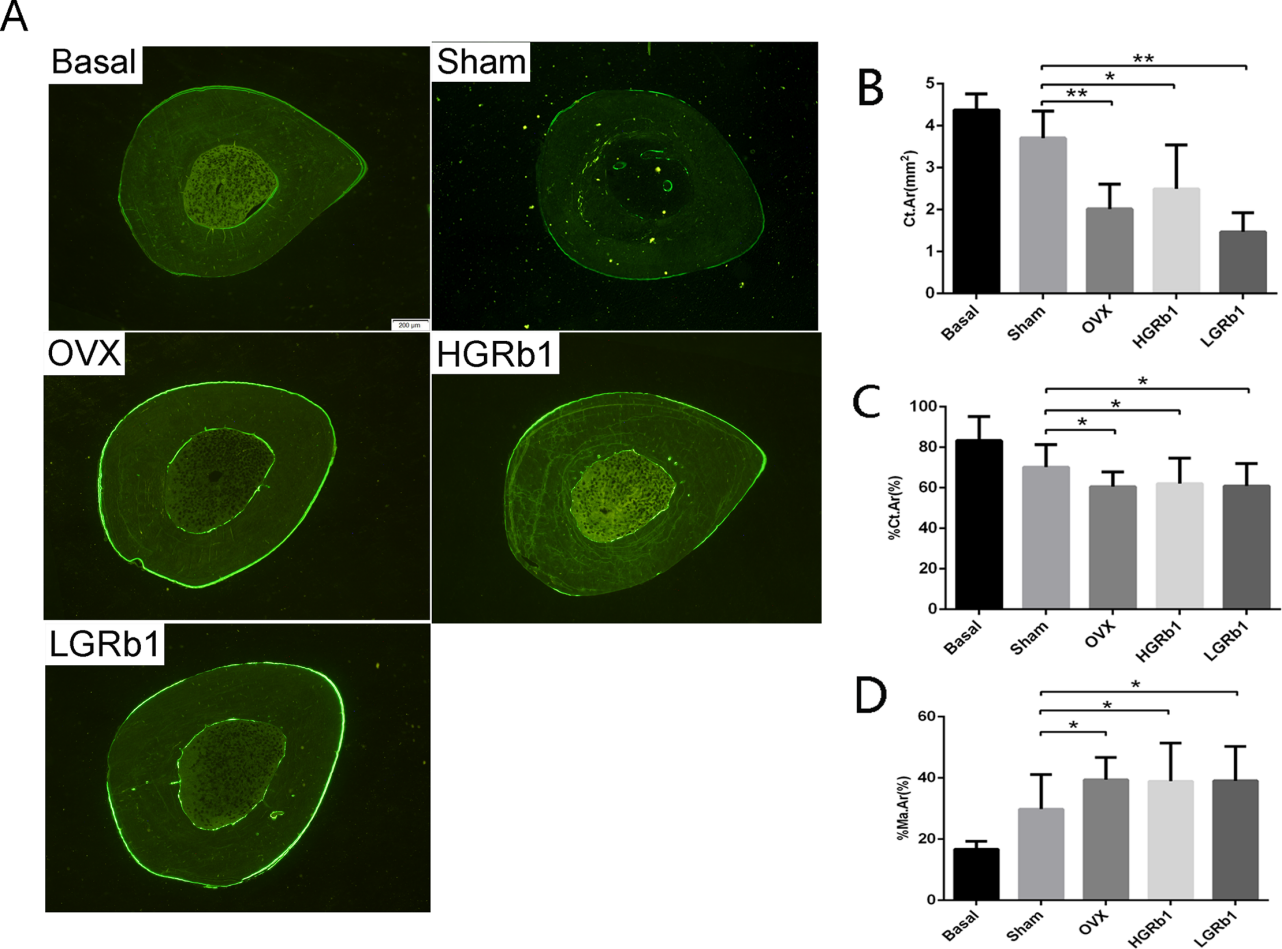
Representative transverse section of distal tibia fluorescence micrographs and histomorphometry analysis. (A) The 8 μm transverse sections of the distal tibia were used for histomorphometric measurements. (B) Ct.Ar (C) %Ct.Ar (D) %Ma. *P < 0.05 compared with Sham.

We measured the Ct.Ar, %Ct.Ar, %Mt.Ar (Fig 8), %L.Pm, MAR, and BFR/BS (S5 Fig) of the periosteum and endosteum in transverse sections of the distal tibia. As the data show, there were non-significant changes in %L.Pm, MAR, BFR/BS of the periosteum and endosteum between the OVX and sham rats, whereas there were significant reductions (P < 0.05) in Ct.Ar and % Ct.Ar and significantly increasing %Mt.Ar(P < 0.05) in OVX rats. Similarly, GRb1 did not prevent bone loss in the cortical bone of the distal tibia in OVX rats.

## 4. Discussion

Previous studies have found that GRb1 has a positive effect on osteoblast differentiation and inhibition of osteoclast differentiation[24,31–32]. Most researchers have reported that GRb1 promotes osteogenesis of osteoblast and inhibits osteoclastogenesis of osteoclasts. Luo Zhi-jun[32] found that GRb1 affects the proliferation and osteogenic differentiation of human adipose-derived stem cells.

MSCs recently have become a new highly studied approach to tissue regeneration. Adult MSCs isolated from different tissues have been studied for their use in musculoskeletal tissue regeneration, which could be extremely valuable for treating patients with musculoskeletal diseases and injury[33–34].

Therefore, we hypothesized that GRb1 actually enhances osteogenesis by stimulating MSCs and performed an animal experiment to investigate whether GRb1 has a positive influence on estrogen-deficiency bone loss. First, we investigated the role of GRb1 on cell viability via MTT and found that GRb1 can improve cell viability at a dose of 10^−8^–10^−4^ M. Additionally, we measured the anti-oxidative effect of GRb1 on H_2_O_2_-induced MSCs and found that 10^−6^ M GRb1 can significantly decrease oxidative damage of H_2_O_2_-induced MSCs, which suggests that GRb1 can act against estrogen-deficiency-induced oxidative stress.

In addition, we measured ALP activity in MSCs treated with GRb1 compared with controls to evaluate the role of GRb1 on ALP, a marker of early osteoblastic differentiation. The expression and secretion of ALP increased, accompanied by increasing osteoblastic differentiation, suggesting ALP plays an important role in bone formation[35]. Our study indicated that GRb1 remarkably increased ALP activity in MSCs at a concentration of 10^−6^ M and promoted calcium foci formation, which is a marker of mineralization.

Furthermore, we measured differences of mRNA expression in some bone matrix factors in MSCs treated with GRb1, including OPN, Runx2, ALP, and OCN. Runx2 directly regulates osteoblast specific gene expression by its specific DNA binding to the osteoblast specific Cis activating element OSE2 (ACCACA)[36], including osterix (Osx). Thurner [37] found that OPN knockout mice have significantly reduced bone fracture resistance, because OPN has some common characteristics with osteoporosis and may be a useful biomarker for osteoporosis. It is closely related to hydroxyapatite in bone tissue and participates in the regulation of bone calcium deposition. Osteocalcin (OCN) is a kind of vitamin K-dependent calcium binding protein secreted by mature osteoblasts, which mainly presents during the mineralization formation stage, so it is considered to be one of the mineralization markers in the osteoblast differentiation period[38]. Overexpression of Runx2 can up-regulate expression of OCN, while Osx, a down-stream gene of Rinx2, can regulate the expression of ALP and OPG. Our results indicate that GRb1 can improve the expression of ALP (P < 0.05) and OPN (P < 0.05), whereas it has no effect on Runx2. Combined with the ALP activity assay results, we concluded GRb1 enhanced ALP activity through up-regulating the expression of ALP. GRb1 can induce MSCs to differentiate into osteoblasts and improve mineralization. Based on the lack of effect on the expression of Runx2, we doubt that GRb1 has any influence on Runx2 phosphate instead of Runx2 because Runx2 phosphate can stimulate the expression of Osx[39–40]. There was a limitation to our study that we should have measured the mRNA expression of Osx.

The ovariectomized model is a classic model of osteoporosis that can mimic the state of postmenopausal osteoporosis in humans. Estrogen deficiency after ovariectomy results in increasing levels of reactive oxygen species, osteoclast activation and bone resorption, presenting with a high turnover type of osteoporosis.

In our experiment, the weight of ovariectomized rats continued to rise significantly in the 12 weeks after surgery. Compared with the sham operated group, there was an upward trend, but there was no statistical significance to the difference. We speculated that although ovariectomy leads to fat accumulation in rats, the weight of increased fat does not compensate for the loss of bone weight. It is well known that ovariectomy results in elevated levels of oxidative stress in rats[40–42]. In our experiment, the levels of SOD and GSH in the serum of ovariectomized rats decreased rapidly, meaning a decrease in anti-oxidant capacity in the ovariectomized rats. Generally, the hormone levels drop after ovariectomy, resulting in significant atrophy of the uterus, decreased uterine wet weight, and decreased endometrial thickness[43], and our present experimental results are consistent with this. Dyslipidemia was found in the OVX rats in our study, most likely as a result of altered energy metabolism caused by estrogen deficiency[44–45].

Our experiments showed that ovariectomy caused severe bone loss and severe destruction of bone microstructure in rats. Although the double fluorescence state showed that ovariectomy accelerated the growth of the trabecular bone, it was still resistant to the rapid loss of bone mass. Similarly, the bone density of the femur decreased, and the bone biomechanical properties worsened, such as increased fragility and diminished load-bearing capacity.

Based on experiments in vitro, we found that GRb1 can improve osteogenesis of MSCs, which have the potential for differentiation, but the results from our animal study showed something different. We first suspected that GRb1 had no apparent effect on osteogenesis in OVX rats, probably because the dose of GRb1 was not high enough. In the in vivo experiments, the drugs will be partially metabolized in the circulatory system, preventing some of the GRb1 from participating in enhancing osteogenesis. Liu Yang [46] found that even just a few molecules of GRb1 in rats can enter the plasma and then be metabolized into an effective product. Second, we think that it may not be sufficient that GRb1 was intraperitoneally injected once daily. According to the previous research[47], GRb1 will be entirely metabolized after existing in the plasma for 2 hours. Therefore, it may be necessary to repeat the administration of GRb1 more frequently. In the future, attention should be paid to the distribution and excretion of drugs in pharmacokinetics, which is the basis of dosage, and sustained-release formulations can be considered. Third, it is possible the bone mass was so rapidly lost in this high turnover type osteoporosis model that GRb1 is ineffective against it. Additionally, it is obvious that bone formation of rats in the femur and tibia is increased after ovariectomy. Bone formation is conditioned so that it cannot be unlimited even when using drugs that have a positive effect on MSCs. We think that it is necessary to prove the effect of GRb1 on OP by using a different OP model.

## Supporting information

S1 Fig HE stain of adipocytes of proximal tibial of rats (40×, scale bar = 200μm; 200×, scale bar = 50μm). (PDF)

S2 FigEffects of GRb1 on blood fat and oxidative stress in OVX rats ($\bar{x}\pm s$, n = 8).*P < 0.05 compared with sham.(PDF)

S3 FigEffects of GRb1 on biomechanical properties of the femur in OVX rats ($\bar{x}\pm s$, n = 8).*P < 0.05 compared with sham.(PDF)

S4 FigRepresentative proximal tibia fluorescence micrographs and histomorphometry analysis ($\bar{x}\pm s$, n = 8).*P < 0.05 compared with sham.(PDF)

S5 FigRepresentative transverse section of distal tibia fluorescence micrographs and histomorphometry analysis ($\bar{x}\pm s$, n = 8).*P < 0.05 compared with sham.(PDF)
