# Supplementary figures and images for: Ginsenoside Rb1 does not halt osteoporotic bone loss in ovariectomized rats
Source: PLoS One. 2018 Sep 13;13(9):e0202885. doi: 10.1371/journal.pone.0202885 (PMC6136715; doi:10.1371/journal.pone.0202885)

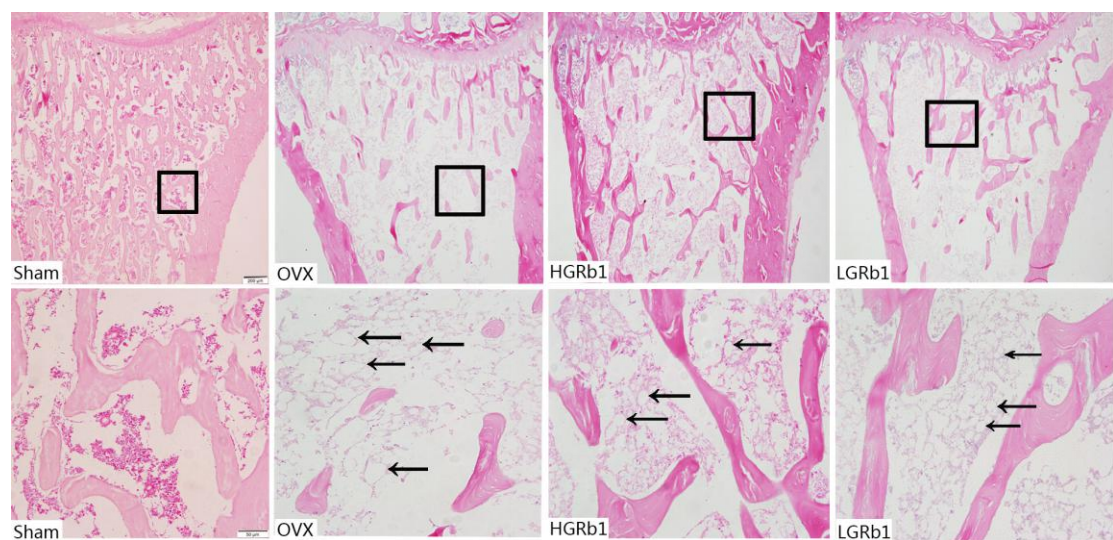

HE stain of adipocytes of proximal tibial of rats  
 ( 40×, scale bar=200μm; 200×, scale bar=50μm)

Supplement: S1 Fig — (PDF) [file pone.0202885.s001.pdf]
