# Supplementary material for: Ginsenoside Rb1 does not halt osteoporotic bone loss in ovariectomized rats
Source: PLoS One. 2018 Sep 13;13(9):e0202885. doi: 10.1371/journal.pone.0202885 (PMC6136715; doi:10.1371/journal.pone.0202885)
Supplement: S2 Fig — *P < 0.05 compared with sham. (PDF) [file pone.0202885.s002.pdf]

Table 1. Effects of the GRb1 on blood fat and oxdative stress in OVX rats（x±s，n=8）

	TC	TG	HDL	LDL	GSH	SOD
**Basal**	1.51±0.254	0.970±0.209	1.39±0.32	0.0587±0.023	8.88±1.18	0.472±0.0280
**Sham**	2.07±0.328	1.23±0.196	2.01±0.186	0.133±0.052	10.6±1.30	0.487±0.0511
**OVX**	3.28±0.606*	1.74±0.307*	1.33±0.268*	0.224±0.043*	9.31±1.10*	0.408±0.0213*
**HGRb1**	2.81±0.520*	1.72±0.139*	1.37±0.268*	0.208±0.058*	9.77±0.918*	0.449±0.0356*
**LGRb1**	2.76±0.497*	1.74±0.259*	1.32±0.218*	0.227±0.047*	9.04±1.28*	0.444±0.0411*

*P<0.05 compared with Sham
