# Supplementary material for: Ginsenoside Rb1 does not halt osteoporotic bone loss in ovariectomized rats
Source: PLoS One. 2018 Sep 13;13(9):e0202885. doi: 10.1371/journal.pone.0202885 (PMC6136715; doi:10.1371/journal.pone.0202885)
Supplement: S3 Fig — *P < 0.05 compared with sham. (PDF) [file pone.0202885.s003.pdf]

Table 2. Effects of the GRb1 on biomechanical properties of femur in OVX rats (x±s,n=8)

Group	Maximum load/N	Break load/N	Ealstic load/N	Rigidity coefficient/N/mm
**Sham**	126.96±8.55	126.64±8.54	96.68±5.34	327.17±32.32
**OVX**	112.63±11.42*	111.21±10.59*	92.93±8.52	303.42±50.15*
**HGRb1**	118.88±6.58*	114.97±5.05*	94.76±5.44	314.28±20.97*
**LGRb1**	113.53±9.52*	115.97±3.72*	94.11±4.90	308.19±19.72*

*$P<0.05$ compared with Sham
