# Supplementary material for: Ginsenoside Rb1 does not halt osteoporotic bone loss in ovariectomized rats
Source: PLoS One. 2018 Sep 13;13(9):e0202885. doi: 10.1371/journal.pone.0202885 (PMC6136715; doi:10.1371/journal.pone.0202885)
Supplement: S4 Fig — *P < 0.05 compared with sham. (PDF) [file pone.0202885.s004.pdf]

Table 3.Representative proximal tibia fluorescence micrographs and histomorphometry analysis.（x±s，n=8）

Group	%Tb.Ar (%)	BFR/TV (%/yr)	BFR/BV (%/yr)	BFR/BS (μm/d*100%)	MAR (nm/d)	%L.Pm (%)
Basal	24.97 ±5.18	174.5 ±42.18	383.32 ±209.10	265.85 ±26.89	177.46 ±34.25	37.32 ±9.49
Sham	22.38 ±6.97	243.04 ±22.99	248.26 ±31.54	243.04 ±102.99	303.99 ±138.05	49.14 ±22.44
OVX	6.80 ±3.73*	488.68 ±23.80*	497.01 ±177.58*	218.26 ±41.54	541.31 ±116.67*	67.01 ±15.98
HGRb1	9.80 ±7.46*	364.00 ±24.99*	475.09 ±210.76*	190.26 ±31.35	499.37 ±73.80*	50.46 ±18.04
LGRb1	9.34 ±1.60*	384.48 ±43.36*	474.07 ±280.07*	202.81 ±51.53	437.82 ±91.03*	58.73 ±47.28

*P<0.05, ** P<0.01 *vs* Sham
