# Supplementary material for: Ginsenoside Rb1 does not halt osteoporotic bone loss in ovariectomized rats
Source: PLoS One. 2018 Sep 13;13(9):e0202885. doi: 10.1371/journal.pone.0202885 (PMC6136715; doi:10.1371/journal.pone.0202885)
Supplement: S5 Fig — *P < 0.05 compared with sham. (PDF) [file pone.0202885.s005.pdf]

Table 4. Representative transverse section of distal tibia fluorescence micrographs and histomorphometry analysis. ($x \pm s$,n=8)

Group	E-BFR/BS (μm/d*1000)	E-MAR (μm/d)	%E-L.Pm (%)	P-BFR/BS (μm/d*1000)	P-MAR (μm/d)	%P-L.Pm (%)
**Basal**	22.63±17.21	1.68±1.05	31.65±10.27	8.64±5.95	2.78±1.04	56.52±7.48
**Sham**	23.91±2.99	1.99±0.56	42.13±11.65	11.00±1.97	3.40±2.65	40.11±11.66
**OVX**	28.13±12.94	2.49±0.86	48.25±11.06	13.66±5.93	3.13±2.33	40.66±20.01
**HGRb1**	19.87±15.07	2.45±0.76	48.29±10.40	9.38±6.83	3.38±1.95	45.81±25.31
**LGRb1**	16.08±5.28	2.05±0.54	40.80±14.63	11.64±7.37	3.55±1.49	42.77±19.92

$^{*}P<0.05$ compared with Sham
